# Improving Student Support in Professional Placement Learning: Findings from the South West Peninsula Pilot of a New English National Placement Quality Assurance and Enhancement Process

**DOI:** 10.2174/1874434600802010021

**Published:** 2008-03-18

**Authors:** Graham R Williamson, Val Heath, Liz Ballantyne, Lynne Callaghan, Daniel Webster, Claire Hunter

**Affiliations:** Lecturer, Adult Nursing, Faculty of Health and Social Work, University of Plymouth, Devon, UK

**Keywords:** Quality assurance, healthcare education, placement learning, student support.

## Abstract

English stakeholder collaboration has resulted in a new quality assurance process for non-medical health and social care placement providers and higher education institutions. This study aimed to discover the impact on student support that taking part in a pilot had on participating placement areas. Using a questionnaire survey with longitudinal follow-up one year later, we found that placement staff valued the opportunity to review and improve student support practices. This was still in evidence a year later where the pilot was described as giving the opportunity to provide evidence of aspects of student support practice; communicating and changing or developing aspects of that practice. Benefits accrued from interdisciplinary working in sharing and collaborating with other professions and organisations. Such activity could enhance clinical support staff activities and facilitate strategic partnerships between placement providers and higher education institutions.

## INTRODUCTION

Globally, there is a drive to assure the quality of education provision including healthcare education, which has seen the establishment of the International Network for Quality Assurance Agencies in Higher Education (INQAAHE), which published Guidelines for Good Practice for external quality assurance agencies [1]. The European Network for Quality Assurance in Higher Education (ENQA) was established to promote greater harmonization of values and good practice in higher education in relation to quality assurance agencies. Their report [2] advocates that institutions develop and implement formal strategies for continuous quality enhancement, to include a role for students and stakeholders such as employers. This agreement covers 40 European countries; principles include autonomy for individual institutions, and subsidiary (meaning review processes should reflect national needs and requirements). In the United States, the regulatory picture is different, without central government involvement. ‘Accreditation’ of higher education providers is by private, non-profit organisations designed for specific purposes. Thus quality assurance is as decentralized and complex as is the higher education sector, with approximately 6,500 accredited degree-granting and non-degree-granting institutions in public and private sectors including healthcare professions [3].

In England, the Department of Health (DH) has been working closely with education commissioners and providers (including higher education institutions [HEIs] and placement providers); the Nursing and Midwifery Council (NMC; the body responsible for holding UK nurses’ registrations, maintaining standards and protecting the public, see www.nmc-uk.org/), the Health Professions Council (HPC; which carries out similar functions for other healthcare professions except medicine, see www.hpc-uk.org/), students and service users, to develop one shared framework for healthcare education that is robust and meaningful. This is intended to reduce the administrative burden on education providers and has resulted in the Partnership Quality Assurance Framework for Healthcare Education in England (PQAF [4, 5]). Part of this is a new process for quality assuring and improving healthcare education and placement learning in England, which is being developed under the direction of Skills for Health (the UK government agency responsible for ensuring that those working in the sector are equipped with appropriate skills to support service development and delivery; see www.skillsforhealth.org.uk). This was known initially as OQME (short for Ongoing Quality Management and Enhancement), and has now become EQuIP (Enhancing Quality in Partnership [6]), and is likely to be an important development for those working in National Health Service (NHS) Trusts, the independent sector, in social care, and in higher education institutions where health and social care students’ programmes of study include practice placements: this new process may become the method by which healthcare educational provision and practice placements are quality assured, replacing or at least significantly altering current processes including Quality Assurance Agency major review, Strategic Health Authority contract monitoring and professional bodies’ (including NMC and HPC) quality assurance procedures for Higher Education Institutions [7,8].

United Kingdom (UK) healthcare education emphasises the importance of learners achieving clinical as well as academic competencies so that students are ‘fit for practice’ on qualification for their professional awards [9]. Recent governmental and professional bodies’ policies emphasize the importance of clinical experience for healthcare professionals [10,11,12], particularly as in the UK half of nursing students’ time in pre-registration programmes takes place in practice settings, where a large amount of formal and informal learning and socialization occurs [13]. Despite the operationalisation of such guidance there are still issues of concern about placement learning. Recent large increases in student numbers have raised questions about placement areas’ abilities to provide learning environments of sufficiently high quality for students, meaning that some institutions programmes did not fully met all the Quality Assurance Agency precepts [14, 15]. In nursing, it has long been theorized that education support staff such as lecturer practitioners and practice educators could provide a solution to many placement learning problems and close the ‘theory-practice gap’ between higher education institutions and practice settings [9,16], but it is now apparent that such roles are neither completely effective nor unproblematic, particularly where managerial support and clear objectives are lacking [16,17].

In terms of clinical practice facilitation, McNamara [18] identifies three essential elements as important for clinical placement facilitators: their role as a dedicated support for students; establishing, maintaining and developing the clinical teaching partnership between staff and students; and ensuring safe practice. McNamara [18] concludes that optimizing students’ clinical learning is a shared responsibility between many staff, but benefits from the availability in clinical settings of a dedicated clinical placement facilitator. This is supported by Henderson*, *Heel and Twentyman [19], who found that effective preceptor activity is a clinical placement strategy that provides adequate psycho-social support for students, although there are more effective methods for supporting students in clinical practice.

Latterly, rather than an emphasis on roles and functions of support staff there has been a focus on how placement learning can be quality assured across multiple professions, how review processes can be streamlined and simplified [20], and how effective, local action can be taken to ensure that improvements are implemented. Research with students [21] highlighted how essential practice experience is in nursing, but also how variable it can be. Good communication was seen as very important amongst health professionals and educational sites with key roles being ward managers, mentors and link tutors; and there was a general recognition that clear quality assurance mechanisms offered the opportunity to improve student support practice and thus to improve student experiences and outcomes. Similarly, Henderson, Heel and Twentyman [19] argue that strategic partnerships and open communication between healthcare organisations and tertiary institutions are vital in ensuring that student support staff function effectively. These developments have brought about increased staff satisfaction through their greater involvement in students’ placements, in turn leading to improved satisfaction and outcomes for the students.

In this new quality assurance approach, from which OQME and its recent iteration EQuIP have been developed, key stakeholders will share a framework of standards for monitoring and programme review that includes learning taking place in both campus and practice-based settings, so that a new partnership between HEIs and practice placement providers emerges [6, 22]. A key principle of this new national process is that it should be multi-professional, and agreement is currently being sought between HPC and NMC and other sector regulatory bodies on underlying principles; the burden on practice placement and education providers should be minimised, saving time and reducing duplication of effort. There is to be a new emphasis on quality enhancement as much as quality assurance, parity of practice-based learning with academic education [6,7], with standards or ‘requirements’ (for HEIs, placement providers and some joint ones, see Table **1** for their current iteration) developed in a series of stakeholder consultation exercises [6,7,22].

A quality review process between local stakeholders will involve self-evaluation, action planning, dissemination of good practice, risk management assessment, independent external verification and scrutiny, and finally, outputs (statistical and qualitative, analytical statements ‘owned’ by all contributors). A final report for will be produced by learning providers and commissioners, for wider publication.

This paper outlines a research study that was undertaken in three counties in the South West of England, where a new quality assurance and enhancement (OQME) data base was developed in collaboration between local HEIs and placement providers in a partnership between Skills for Health (who retain the intellectual property rights) and universities with responsibility for placing healthcare students in the South West Peninsula. This pilot activity took place in spring 2005 to investigate aspects of the OQME data base using a Microsoft ACCESS database originally developed by Teignbridge District Council. The pilot was a comprehensive evaluation of technical and procedural aspects of the data base. As the larger study aim was met and data fully evaluated elsewhere [23], this study discusses only issues of student support; a discreet and important topic in itself and one of interest to purchasers, to placement providers at organisational, unit and individual level, and to HEIs.

## MATERIALS AND METHODOLOGY

### Aims and Objectives

The aim of this study was to discover the impact on student support activity that taking part in the OQME pilot had on those placement areas that participated. Objectives were to discover if, and how, student support policies and practices were developed in these placement areas as a result of the pilot activity.

### Study Design

This study was a longitudinal design. After taking part in the data base pilot (May-June 2005), evaluative data were collected in a questionnaire, from which issues of student support will be reported. Further follow-up to assess the longer term impact on student support was undertaken by telephone interview in summer 2006.

### Data Collection

A questionnaire was chosen as an appropriate method for collecting data in the initial phase of the study for pragmatic reasons; namely, the ease and simplicity it gave, with a minimum of burden to placement area staff who had been involved with the time-consuming OQME pilot process. The questionnaire contained 23 open ended questions designed by the researchers, allowing for the maximum of flexibility in responses from participants. Telephone interviews were chosen for the follow-up one year later to obtain an in-depth picture of how enduring were any changes to student support practices, and again for pragmatic reasons of ease of access to busy clinical staff who would be unlikely to attend timetabled interviews on a University campus away from their clinical bases: telephone methods have been noted to improve participation rates, and to be particularly useful in accessing participants in dispersed geographic locations [24], and were thus ideal for our purpose in recruiting across three rural counties in South West England. A schedule of questions was developed by the researchers, aimed at eliciting participants’ views concerning whether student support policies and procedures were altered by taking part in the OQME pilot and if these developments were enduring one year later (see Table **2**). These interviews lasted approximately 20 minutes, and were transcribed and analyzed.

### Participants and Sampling

In spring 2005, nine clinical placement providers and three HEIs in the South West Peninsula of England were invited to take part in an exercise piloting a Microsoft Windows ACCESS electronic database for the OQME standards in their prototype format. Placement providers were chosen as representative of the diversity of local placement areas. The clinical areas were a Cardio Respiratory Unit, an Orthopaedic Ward and a Radiography Department from a Healthcare NHS Trust, an Intermediate Care Unit, a Speech and Language Therapy Department and a Health Centre from a Primary Care Trust, and two hospices and a Macmillan nurse (although she did not complete due to IT problems) from a Hospice and Palliative Care service. Three education institutions participated in the self-assessment of the HEI standards and the joint standards with placement provider units. These were HEI 1 with Healthcare NHS Trust placements in their pre-registration nursing programme, HEI 2, a College with a Speech and Language Therapy Department and Primary Care Trust placements in their pre-registration Allied Health Profession Programme, and HEI 3, a University with Hospice placements in their Post-qualifying Partnership Palliative Care Module. In each setting, a senior member of staff with a responsibility for education and student support participated in the study by completing the ACCESS data base and emailing it to the Health Authority for collation [23], completing the questionnaire and later taking part in the telephone interviews. As two of this study’s authors (GRW and VH) from one HEI participated in data base and questionnaire completion they were not interviewed by telephone for the longitudinal follow-up.

### Ethical Issues

The questionnaire arm of the study audited the pilot and so did not require formal ethical approval. The telephone arm of the study was submitted for approval by the NHS Combined Research Ethics Committee (COREC, a body charged with ethical approval of research taking place in more than one NHS site in England) but was also deemed by them to be audit and their approval to undertake the study was given on that basis. Informed consent was secured verbally after the study aims and objectives and issues of data collection and analysis were explained. Participants were assured that their confidentiality and anonymity would be respected, and that they had the right to withdraw at any time without prejudice. Telephone interviews were undertaken by a research fellow and a research assistant at the University’s Centre of Excellence in Professional Placement Learning (CEPPL) ensuring that the principal investigator (GRW) was sufficiently distanced from the research and participants to avoid potential biases.

### Data Analysis

Data from the telephone interviews were collected as responses to the open-ended questions and overarching themes produced as a simplification of these data. Data from the telephone interviews were analysed using Miles and Huberman’s [25] framework. This took place in three forms: data reduction, data display and drawing and verifying conclusions. Data reduction took place throughout the analysis, and involved summarizing, coding (labeling and categorizing data) and memoing (theorizing and writing-up ideas about codes and their relationships). Data display involved organizing and compressing data at all stages of the analysis; an essential activity given that qualitative data is voluminous. Thirdly, conclusions were drawn and verified: these were not finalized until all the data are collected, although ideas developed throughout the analysis [26].

### Rigour and Data Analysis

It is essential that qualitative research is rigorous, trustworthy and credible [26, 27]. In order to ensure that this study met these criteria, as well as the data analysis steps described above, three researchers (GRW, LC and DW) analysed the questionnaire findings and the interview transcripts independently and compared analyses. Any differences were resolved through discussion of key themes and their interpretation. Findings were compared and contrasted to get a picture of the extent and nature of changes and developments that occurred in the participating placement provider organisations as a result of taking part in the pilot, with questionnaire data used to give an initial picture of the impact and telephone interview data used to assess the longitudinal impact.

## RESULTS

The response rate to the questionnaire element was 100% (n=12). Telephone interviews were conducted with seven of these participants, of which one recording was ineligible.

### Questionnaire

Findings in the form of themes generated from the questionnaire element relating to student support are listed in Table **3**. Taking part in the pilot was noted as giving the opportunity to review or reflect upon, and improve practices in regard to student support and the resources available with which to do this. There were also benefits accruing from interdisciplinary working, in the sense of sharing and collaborating with other professions and organisations.

Anticipating the main long-term benefits of OQME, respondents believed that it would enhance the placement experience for students, provide evidence for the quality of placements, and improve the relationships between HEIs and placements because of the new requirement to share and agree the joint standards of the OQME document. One potential gain noted was that OQME would enable sharing of best practice.

Overall, participants enjoyed being part of the OQME data base pilot, believing that it gave them the opportunity to focus on improving students’ placement experience, as well as an opportunity for collaborative working. Two quotes illustrate participants’ thinking concerning how taking part in the pilot had impacted on student support. Both indicate that a major gain for clinical placement areas involved in OQME was the opportunity to discuss, collect and collate existing information, policies and practice concerning student support:

‘It was a delight to see how much good practice there was and for us to be able to congratulate ourselves on all the positive work we are achieving with student support.’

Another participant noted that the OQME process was: ‘firmly rooted in what we are doing and need to do to improve and enhance placement quality and opportunities’, showing a similar focus on identifying good practice and the benefit this might have for students.

### Telephone Interviews

Analysis of the data revealed four themes: evidencing, sharing and communication, changing practice, and operational aspects of OQME. Each of these themes is presented below, illustrated by quotes from the data (summarized in Table **4**).

### Evidencing

Participants were generally positive about the implementation of OQME in their placement areas. One of the strongest themes to emerge from the data illustrating this positive perception was how OQME enabled staff in placement areas to ‘evidence’ concerning what student support activities had been carried out. OQME therefore provided a structure for assembling evidence of such procedures. For example, Participant 1 reflected on the procedures in place prior to OQME, and how the new system enabled clarification of evidence:

‘It was very piecemeal and… [staff] knew what needed to be done but having that… clear process to work [with]... made them think about what they were doing and how to evidence it how we were [previously] evidencing things was very piecemeal.’ (Participant 1).

Several participants highlighted the capacity of OQME to enable and to emphasize the importance of documenting and evidencing procedures within their placement areas. Further, one participant stated the usefulness of increased documentation in providing evidence of student support where the level of such support can be disputed:

‘…It covered my back really…if students don’t feel supported…and we can show that they actually are…we are doing all that is required of us…’ (Participant 3).

Therefore, although it was agreed by participants that the OQME pilot did not change the policies that were already in place in the pilot placement areas, it did provide a framework that allowed existing processes to be measured. Furthermore, by being able to observe these processes in a structured way, participants were able to feel confident about the efficacy of their current practices concerning student support:

‘…Probably the most valuable thing from my point of view looking at it now, was the issue of having some sort of measurable [sic] tool that looked at what we’ve already got … in place at the moment…at that stage there was no real direction, and actually it was reassuring to find that a lot of the things that… they were looking for were actually already in place and weren’t actually too difficult to find. It was probably reassuring that there was already good practice going on, really…’ (Participant 7).

In some instances, however, although OQME did not have a direct impact on *policy*, there was some impact with regard to student support *practice*, which was stimulated by the evidencing processes discussed above. Therefore, by engaging with a tool that enabled comprehensive evidencing of procedures and processes in place, any gaps in this evidence were highlighted as were subsequent changes in practice required.

### Sharing and Communication

The second theme regarding positive aspects of involvement in the pilot was the development of sharing and communication of practices both between placement areas and HEIs and also within the placement areas. By creating closer links between placement providers and universities, lines of communication were opened which further enhanced opportunities for sharing existing best practice captured by OQME:

‘…They were certainly starting to work more closely with some of the universities…as some of them had been a little bit distant. But, they were getting more involvement and more feedback through universities, as a result trying, actually trying to do the work for the OQME they realized that they needed to communicate more to get this kind of thing happening…’ (Participant 1).

Another participant noted that: ‘It was very useful…to do the joint standards and the discussion …for the future.’ (Participant 4).

Therefore, the implementation of OQME both encouraged greater communication to take place and helped participants to realize the importance of such communication and sharing. Similarly, the process of ‘evidencing’ work (discussed above) in itself necessitated cooperative working. For example, one participant found great value in a benchmarking exercise undertaken by herself and a colleague from another profession:

‘…We actually found it a really helpful exercise and because we did it jointly… [we were] able to do a lot of unifying of paperwork together…and [it] actually helped us to share the idea that we had [about our] best practice. So that was really helpful… it was very helpful as a tool, and to share best practice…’ (Participant 6).

Therefore, taking part in the OQME pilot and undertaking procedures involved in its operation provided, for the majority of participants, a rationale for both internal and external cooperation and dissemination.

### Changing Practice

The two themes presented above show how, in general, engaging in the OQME pilot underlined the need for the comprehensive documentation of evidence, for improved communication in terms of student support practice, and for dissemination within and outside placement areas. In some cases this need emphasized that a change in practice was necessary in order to fulfill the requirements of OQME. As discussed above, although participants did not believe that any policy changes took place as a result of the use of OQME, certain practices were adapted or changed as a result:

‘…it’s about strengthening induction materials and individual units sort of learning from best practice, if you like…picking up on that and trying to improve what they had…so that’s process or procedure but in terms of policy, no…’ (Participant 2).

Further, as in the example below, in some cases new resources were created as a direct result of participation in the pilot. For example, the production of an action plan (below), initiated due to the use of OQME, instigated a change in practice with the use of a checklist benefiting both supervising staff and their placement students.

‘I suppose there’s just the longer term impact really because I think the useful thing about being involved was, um, actually being able to get an action plan for the department that’s been something we’ve just been able to use in our annual service plan which has been really useful… one of the things we did from the action plan was develop a general…checklist just for the first day for the supervising clinicians to use…’ (Participant 4).

Another participant acknowledged that since the commencement of the OQME pilot the tutorial time of ‘return to practice’ students had been increased, therefore showing a direct change in practice in response to OQME:

‘…Probably the main thing I think really - especially from the return to practice side of it… was we’ve increased the tutorial time…probably over and above what the University was originally saying’. (Participant 7).

### Operational Aspects of OQME

Participants spoke about a number of technical issues related to the implementation and operation of OQME in their placement areas. These issues ranged from organisational ones concerning implementing the OQME pilot process and the resources involved, to the IT skills and workload of staff utilizing the tool. Most of these technical issues were met and dealt with in the initial stages of the application of the tool, for example:

‘…The biggest problems were technical... [these] are now being sorted out. Since then the whole computer system has been upgraded. That was half the problem: the actual resource itself was running on a much higher spec[ification] than… what we had at the time. We produced a lot of work towards the end, because we were waiting for the upgrade before we could actually do it’. (Participant 1).

Another participant stated difficulties with the set up of the system, although once installed found it to be user friendly despite initial anxiety regarding her level of necessary IT skills:

‘I did take it on rather, sort of, in trepidation, because I didn’t think my IT skills were wonderful for doing something like that, but other than the initial hiccup we had with just getting the system set up which was an NHS and organisational problem the system in place could use the data base, it was really very easy to use and user friendly’. (Participant 4).

Although it appeared that, overall, implementation difficulties were relatively easily resolved, participants did comment that using the tool to document the evidence that they found so useful was a repetitive process:

‘…I think the computer system was…quite…complex really…a bit repetitive…yeah I think some of the questions on it were very repetitive.’ (Participant 3).

Further, despite the benefits of the introduction of the tool that were stated by participants, both the implementation and day-to-day operation of OQME necessarily added pressure to normal workloads:

‘I think the IT challenges usually were a headache because we were left with very, very little time to do it, and that was a pressure because obviously that’s additional to the normal workload and…there’ll be lots of other pressures then.’ (Participant 4).

In summary, it is clear that a high degree of consensus existed between the findings from the questionnaire administered immediately after the OQME pilot in summer 2005 and the more in-depth telephone interviews carried out a year later in 2006. In the questionnaire, taking part in the OQME pilot was noted to give placement areas the opportunity to review, or reflect upon, and to improve student support practices and the resources available, and this was still in evidence a year later where the pilot was described as giving the opportunity to provide evidence of aspects of student support practice, sharing and communicating and changing or developing aspects of that practice. There were also benefits accruing from interdisciplinary working, in the sense of sharing and collaborating with other professions and organisations was mentioned in the questionnaire findings, and this was also a key theme in the telephone interviews. In summer 2005, respondents anticipated long-term OQME benefits, believing that it would enhance the placement experience for students; provide evidence for the quality of placements; and improve the relationships between HEIs and placements because of the new requirement to share and agree the joint standards of the OQME document. One significant potential gain noted was that OQME would enable sharing of best practice. All of these areas emerged from the telephone interviews in summer 2006.

## DISCUSSION

The OQME (now known as EQuIP) process of quality assurance for student support is different from a more traditional ‘personal’ approach in which clinical support staff undertake activities face-to-face with learners in their placement areas and have influence in curriculum design and delivery in HEIs. In nursing, such staff have carried role titles such as lecturer practitioners, clinical facilitators and practice educators [9,14]. Benefits to students and organisations including clinical credibility, link activities between education and service, and personal student support and facilitation have been noted in one systematic review of the research literature [16]. However, nationally these roles are busy and complex, demanding special skills to be successful with the potential to be stressful for post-holders [17]. Indeed, whilst a key *raison d’etre *for clinical support staff was to overcome the ‘theory-practice gap’ in nurse education [9,16], this is difficult to quantify and is now widely questioned in the UK [16].

Student support becomes more problematic for clinical support staff without clear objectives or management backing. Indeed, without clear quality assurance mechanisms and good communication [21] student support is likely to be difficult regardless of roles played by clinical practice facilitation staff. This study demonstrates that OXME/EQuIP gives a clear, measurable structure for placement activities including student support, and in conjunction with dedicated placement staff could give authority to improve resources, implement change, share best practice and document activities that will be audited, action plans required and placement areas given the opportunity to demonstrate their successes. These activities could form the basis for the essential strategic partnerships [19] which Skills for Health seek to build [6,7].

### Study Limitations

This was is a small scale qualitative study, which took part in one geographic location in the South West of England, findings are therefore not generalizable, and conclusions and recommendations are therefore tentative. However, the rigorous data collection and analysis steps outlined above lead us to argue that the study findings are trustworthy and credible, potentially transferable to other settings, and certainly of interest to all health and social care education stakeholders including purchasers.

## CONCLUSIONS

This longitudinal follow-up of a pilot activity for the OQME data base has demonstrated that benefits for student support for this activity concern the ability to provide evidence and to document student support practices, the ability to share and collaborate within and outside the placement and to change aspects of student support practice. This study demonstrates that there is potential in the English quality assurance approach (now known as EQuIP) as a means of quality assurance and enhancement for health and social care placement student support activity and that positive aspects are enduring. One key recommendation therefore is that this approach should be implemented nationally. It is possible that a combination of EQuIP and clinical support staff would improve student support for placement learning in health and social care settings by providing a combination of structure and evidence, and by embedding the authority to improve and develop aspects of student support in the culture of organisations, providing a base for strategic partnerships [19]. This activity would be supported by annual health authority monitoring and the necessity of constructing action plans. Such quality assurance and enhancement forms a platform to capture data and enhance HEI and placement provision, and such activity could be linked to European or international quality assurance frameworks or guidance such as those of ENQA [2] or INQAAHE [1].

Clearly, before placement areas are able to benefit from this new strategy, the tool requires successful implementation, and that appropriate information technology (IT) is available to do so. To this end, we recommend that the forthcoming Skills for Health national IT tender for the web-based development of EQuIP needs to establish and take into account the hardware, software and IT skills available to NHS, social care and independent sector staff and organisations, as well as HEIs. There needs to be a focus also by the tendering organisations on training and technical support when EQuIP ‘goes live’ in England. When this occurs, a national research programme should be undertaken demonstrating improvements in student support resulting from the new quality assurance and enhancement activity, and this should build on these qualitative findings with large scale quantitative work.

## Figures and Tables

**Table 1.** EQuIP Requirements

Values Evaluating, maintaining and improving quality Resource management and governance Teaching and learning Student/learner selection, progression and achievement Student and learner support Assessment

**Table 2.** Schedule of Questions for Telephone Interviews

Tell us about your experiences of taking part in the OQME pilot Tell us about your experiences of student support at your practice placement (e.g. student numbers, programmes and professions; learning opportunities, mentorship) Were policies and/or practices concerning student support changed or developed as a result of the OQME pilot? Did the OQME pilot enable you to change or develop any other aspects your student support? Were any other policies and/or practices changed or developed as a result of the OQME pilot? What impact have these developments made in your practice placement?

**Table 3.** Questionnaire Findings Relating to Student Support

**What were the positive aspects of taking part in the pilot?** Review/reflect/improve on student support and resources Positive impact of interdisciplinary working **What do you think will be the main long-term benefits of OQME?** Enhancing the placement experience for students Provide evidence for the quality of placements Improve relationships between Higher Education Institutions and placements Enable sharing of best practice **Have you enjoyed being part of the pilot of the OQME database?** Opportunity to focus on improving students’ placement experience Opportunity for collaborative working

**Table 4.** Four Themes from the Telephone Interviews

‘Evidencing’ Sharing and communication Changing practice Operational aspects of OQME.
